# Possible Role of GnIH as a Mediator between Adiposity and Impaired Testicular Function

**DOI:** 10.3389/fendo.2016.00006

**Published:** 2016-02-03

**Authors:** Shabana Anjum, Amitabh Krishna, Kazuyoshi Tsutsui

**Affiliations:** ^1^Department of Zoology, Banaras Hindu University, Varanasi, India; ^2^Department of Biology, Waseda University, Tokyo, Japan

**Keywords:** GnIH, adipose tissue, testis, GLUT4, GLUT8

## Abstract

The aim of the present study was to evaluate the roles of gonadotropin-inhibitory hormone (GnIH) as an endocrine link between increasing adiposity and impaired testicular function in mice. To achieve this, the effect of GnIH on changes in nutrients uptake and hormonal synthesis/action in the adipose tissue and testis was investigated simultaneously by *in vivo* study and separately by *in vitro* study. Mice were treated *in vivo* with different doses of GnIH for 8 days. In the *in vitro* study, adipose tissue and testes of mice were cultured with different doses of GnIH with or without insulin or LH for 24 h at 37°C. The GnIH treatment *in vivo* showed increased food intake, upregulation of glucose transporter 4 (GLUT4), and increased uptake of triglycerides (TGs) in the adipose tissue. These changes may be responsible for increased accumulation of fat in white adipose tissue, resulting in increase in the body mass. Contrary to the adipose tissue, treatment with GnIH both *in vivo* and *in vitro* showed decreased uptake of glucose by downregulation of glucose transporter 8 (GLUT8) expressions in the testis, which in turn resulted in the decreased synthesis of testosterone. The GnIH treatment *in vivo* also showed the decreased expression of insulin receptor protein in the testis, which may also be responsible for the decreased testicular activity in the mice. These findings thus suggest that GnIH increases the uptake of glucose and TGs in the adipose tissue, resulting in increased accumulation of fat, whereas simultaneously in the testis, GnIH suppressed the GLUT8-mediated glucose uptake, which in turn may be responsible for decreased testosterone synthesis. This study thus demonstrates GnIH as mediator of increasing adiposity and impaired testicular function in mice.

## Introduction

Nutrition has a significant impact on reproductive processes, including steroidogenesis, gametogenesis, early embryonic development, etc. (1, 2). This association is because reproductive activities are energetically expensive, and the brain modulates the reproductive processes according to nutritional availability (3, 4). The reproductive tissues appears to have a number of “nutrient sensing” mechanisms that may link nutrient status to the reproductive system. Glucose is a very important mediator of nutritional effects on reproduction. Blood concentrations are inversely correlated to energy intake (5). Glucose is transported by the family of facilitative glucose transporters (GLUTs), which are involved in hypothalamic regulation (6, 7). Glucose also plays a major role in providing metabolic substrates to germ cells in the gonads (8, 9). GLUTs in cells act as glucose sensors. Glucose availability influences lutienizing hormone (LH) secretion through gonadotropin-releasing hormone (GnRH) system (10). Insulin is a modulator of the metabolic stimulus and plays crucial roles in the relationship between changes in nutritional levels and reproduction (11). The anabolic actions of insulin on peripheral tissues are well established and plasma insulin also serves as a signal of body fat content to the central nervous system (12). Insulin also amplifies the lipogenesis in adipose tissue (13). Glucose is made available in the body by insulin, which helps to lower the level of circulating glucose by promoting its uptake either in adipose tissue or muscle cells through GLUT4 (14). In the gonads, glucose is essential for maintenance of spermatogenesis *in vivo* (15, 16). The isoforms of GLUTs are expressed in the testis (17). GLUT8 appears to be one of the main GLUTs in the testis (18). Furthermore, it has been demonstrated that adequate amount of GLUT is required for proper testicular activity (18). Insulin has a direct effect at the testis level (16). Insulin receptors (IR) are expressed in both somatic cells, such as Leydig, Sertoli, and peritubular cells, and germ cells in the testis of various vertebrate species (19). IR signal through the IR substrate proteins (IRS) (20, 21) plays a role in regulating fertility under normal fed conditions.

Adipose tissue is the main organ in the body that provides a storage site for triglycerides (TGs) and deals with energy homeostasis. Serum glucose is taken up and stored as fatty acid via lipogenesis in adipocytes, whereas the fall in glucose levels stimulates lipolysis, leading to release of TGs/fatty acid. Mature adipocytes synthesize and secrete numerous hormones called adipokines, which are involved in overall energy homeostasis and also modulate reproductive activities. Recent studies have shown a negative relationship between adiposity and testicular function (22, 23). Although a more recent study suggested a strong association between metabolic disorders and infertility (24), the factors mediating the influence of nutrition on reproduction are currently not clearly known and require detailed investigation.

The neural elements within the brain that control nutritional function and those that control reproduction are interconnected. Thus, studies are required to understand how the neural system that affects food intake may impact on reproductive function. As a generalization, neuropeptides that stimulate reproduction inhibit food intake and *vice versa*. Central (neuroendocrine) regulation of both reproduction and nutritional function provides a meas whereby surplus energy or energy deficit can be perceived, and accordingly, food intake and energy expenditure can be modulated (25). In 2000, a novel hypothalamic neuropeptide that actively inhibits gonadotropin release was discovered in quail and termed gonadotropin-inhibitory hormone (GnIH) (26). GnIH has a C-terminal Arg–Phe–NH_2_ (RFamide) motif and acts via GnIH receptor (GnIHR), a new member of G-protein coupled receptor superfamily (GPR147), to inhibit gonadotropin release and synthesis (27, 28). The follow-up studies demonstrated that GnIH acts as a new key player for regulation of reproduction in birds and mammals (27, 28). GnIH is one of the RFamide peptides, and it is also known as RFamide-related peptide (RFRP) in mammals (27, 28). It is known that GnIH acts on gonadotropes in the anterior pituitary and GnRH neurons in the hypothalamus to inhibit gonadotropin release and synthesis via GnIHR (GPR147) in birds and mammals (27–29). Recent evidence further indicates that GnIH operates at the level of the gonads as an autocrine/paracrine regulator of steroidogenesis and gametogenesis in birds and mammals (27–29).

Recent studies suggested an inverse relationship between increasing adiposity with regressive changes in testis (22, 30). The factors responsible for regulating the inverse association between obesity and testicular activity are not yet known. GnIH is shown to have dual function: it suppresses reproductive activity while promoting fat accumulation by acting as an orexigen in birds and mammals (31–33). It is thus possible that GnIH may be the endocrine mediator between nutritional changes associated with adiposity and changes in the reproductive status. If above hypothesis is true, the GnIH should regulate food intake and promote accumulation of fat in adipose tissue; at the same time, it should exert inhibitory effect on testicular function. Therefore, the aim of this study was to determine the role of GnIH as a modulator of both testes (testosterone synthesis) as well as adipose tissue (accumulation of fat) function. This was achieved by investigating the effect of GnIH on changes in nutrients uptake and hormonal synthesis/action in the testis and adipose tissue simultaneously by *in vivo* study and separately by *in vitro* study.

## Materials and Methods

### Animal

Adult Parkes strain male laboratory mice (*Mus musculus*) were obtained from the inbred colony maintained in our animal house. All experiments were conducted in accordance with principles and procedures of the 2002 Animal act, India, and approved by Animal Ethical Committee, Banaras Hindu University. The adult mice (13 weeks old) of nearly equal body weight (approximate body weight = 30 g) were used in this study. Mice were housed under optimum conditions of temperature 24 ± 2°C and humidity 50 ± 5% in a photoperiodically controlled room (12-h light:12-h dark) and were provided with commercial food (Pashu Aahar Kendra, Varanasi, India) and tap water *ad libitum*.

### Chemicals

GnIH or mRFRP-3 (SIKPSAYLPLRF-NH2) was synthesized by Son et al. (34). Insulin was obtained from torrent Pharmaceuticals Ltd., Mehsana, India. Antibodies to IR β-subunit, GLUT4, and GLUT8 were purchased from Santa Cruz Biotechnology (Santa Cruz, CA, USA), and protein kinase B (PKB)/AKT was purchased from GeneScript USA Inc. (Piscataway, NJ, USA). All other chemicals were purchased from Merck, New Delhi, India. The specificity of antibodies is shown in Table 1.

**Table 1:** **Details of antibodies used for Western blot**.

Antibody	Target species	Species raised in; monoclonal/polyclonal	Source	Concentration (used for Western blot)
GLUT4	Human	Rabbit; polyclonal	Santa cruz Biotechnology Inc. (H-61, SC 7938)	1:500
GLUT8	Human	Rabbit; polyclonal	Santa cruz Biotechnology Inc. (N-60, SC 30108)	1:500
Insulin receptor β	Human	Rabbit; polyclonal	Santa cruz Biotechnology Inc. (C-19, SC 711)	1:500
AKT	Human	Rabbit; polyclonal	GeneScript Inc. (A00965-40)	1:200
Actin	β-Actin	Mouse; monoclonal	Sigma A2228, 128K4813	1:2000

### *In Vivo* Study

Mice were injected daily with three different doses (20, 200 ng, and 2 μg/day) of GnIH dissolved in normal saline for 8 days (*n* = 10 per group), intraperitoneally. Mice in the control group received vehicle only. The dose and duration of GnIH was selected based on the previous study (31). Food intake was measured at every 24 h. The animals were sacrificed by decapitation under a mild dose of anesthetic ether, 24 h after the last injection, and blood was collected. Body mass of each mouse was recorded before killing. Testes and adipose tissue were excised out, cleaned, weighed, and kept at −40°C for immunoblot analysis. The adipose tissue accumulated in the abdominal cavity region of the control and treated mice were excised out and weighed. Serum was collected and stored at −20°C untill testosterone assay, glucose assay, and TG assay.

### Food Intake Measurement

All mice were individually housed in standard polypropylene cage with hopper style feeder, for lab blocks keep food waste minimum and wood shavings scattered on the floor. Each mouse was provided with measured amount (25 g) fresh mice feed between 1,000 and 1,100 h daily for 8 days, and the leftover food pellets (excluding fecal matter) were weighed at the end of 24 h. The total food consumed by each mouse every day was calculated by subtracting the weight of leftover food from the total amount of food given. From the individual data, average food consumed by the control and treated group were subsequently calculated. The average food consumption was measured on day first, fourth, and eight of the experiment. Average daily food intake was calculated as sum of the average food intake on day first, fourth, and eight and divided by three. The amount of food intake by each mouse showed not much change up to day third, but from day fourth up to day eighth, mice the treated with GnIH showed marked increase. Generally, the mouse consumed more food during night as comparable to day. The control mouse showed no significant change in food intake throughout the study.

### *In Vitro* Study

#### Testis Culture

Adult testes (*n* = 8 testes) were quickly dissected out and cleaned of any adhered fat tissue in Dulbecco modified Eagle’s medium (DMEM; Himedia, Mumbai, India) containing 250 IU/ml penicillin and 250 mg/ml streptomycin sulfate. The testes were cut into equal pieces (approximate 10 mg in weight) and cultured by the method as described previously (35). Culture medium was a mixture of DMEM (with sodium pyruvate and l-glutamine) and Ham’s F-12 (1:1; *v*:*v*) (Himedia, Mumbai, India) containing 100 U/ml penicillin, 100 μg/ml streptomycin, and 0.1% bovine serum albumin (BSA; Sigma, St Louise, MO, USA). After initial incubation for 2 h at 37°C, culture medium was discarded, and testicular slices (1 per tube) were finally cultured in 1-ml medium containing 10^−10^ and 10^−9^M/ml GnIH along with or without 10 and 100 ng/ml luteinizing hormone (LH) in a humidified atmosphere with 95% air and 5% CO_2_ to maintain pH 7.4 for 24 h at 37°C. The doses of GnIH and LH used for the *in vitro* study were selected from our previous study (31, 36). Testes cultured under these conditions appear healthy and do not show any sign of necrosis. Each treatment group was run in triplicate. This is evaluated by Autospan liquid gold-Lactate dehydrogenase (Surat, Gujarat, India) in culture media after 2 h of incubation at 37°C. Testicular slices were collected at the end of culture, washed several times with PBS, and stored at −40°C for immunoblot study, and media were collected, stored at −40°C until testosterone assay and glucose uptake (35).

#### Adipocytes Culture

White adipose tissue (WAT) collected from abdominal cavity of adult male mouse (*n* = 6) was used to determine the *in vitro* effects of GnIH with or without insulin on GLUT4, IR, and AKT/PKB protein expression in WAT of male mouse. The dose and duration of RFRP-3 was selected based on previous study (37). We assayed these biochemical markers at three doses of insulin. Culture methods for WAT were adopted according to Roy and Krishna (38). Following collection, WAT was quickly cut into pieces in DMEM (Himedia, Mumbai, Maharashtra, India) containing 250 IU/ml penicillin and 250 mg/ml streptomycin sulfate. Pieces of WAT of equal mass were cultured in a mixture of DMEM (with sodium pyruvate and l-glutamine) and Ham’s F-12 (1/1 v/v) (Himedia) containing 100 IU/ml penicillin, 100 mg/ml streptomycin, and 0.1% BSA (Sigma, St Louise, MO, USA). After initial incubation for 2 h at 37°C, the culture medium was discarded, and pieces of WAT were cultured in 1-ml medium containing either 1, 5, or 10 μg/ml insulin or 10 ng/ml GnIH in a humidified atmosphere with 95% air and 5% CO_2_ to maintain pH 7.4 for 24 h at 37°C. Each treatment group was run in triplicate. After culture, WAT was collected, washed several times with phosphate buffer saline (PBS), and kept frozen at −40°C until immunoblot assay, and media were collected, stored at −40°C until glucose uptake (38).

### Immunoblot

The testes and WATs collected at the end of *in vivo* and *in vitro* studies were processed for protein extraction using the method described earlier (39, 40). Western blot analysis was performed as previously described (38). In short, a 20% homogenate (w/v) of adipose tissue was made in suspension buffer containing 0.1M NaCl, 0.01M Tris–HCl (pH 7.6), 0.001M EDTA (pH 8.0), and 10 μg/ml phenylmethylsulfonyl fluoride. The homogenate was centrifuged at 5,000 *g* and at 4°C for 15 min; the supernatant was extracted with an equal volume of chloroform and the aqueous phase was recovered. Equal amounts of proteins (40 μg) as determined by Folin’s method were used for 10% SDS-PAGE. Thereafter, proteins were transferred electrophoretically to a PVDF membrane (Millipore India Pvt. Ltd., Bangalore, Karnataka, India) overnight at 4°C. Membranes were blocked for 1 h with Tris-buffered saline [TBS; Tris 50 mM (pH 7.5), NaCl 150 mM, 0.02% Tween 20] containing 5% fat-free dry milk. The membranes were further incubated with primary antibody (see Table 1) for 1 h in blocking solution. Immunodetection was performed with anti-rabbit IgG conjugated horseradish peroxidase (1:1,000) for 4 h. Finally, the blot was washed three times with TBS and developed with an enhanced chemiluminescence (ECL) detection system (Bio-Rad, Hercules, CA, USA). Similarly, a blot was developed for β-actin (Sigma-Aldrich, India) at a dilution of 1:1,000 as a loading control. Immunoreactive bands were later quantified using Image J software (Image J 1.36, NIH, Bethesda, MD, USA) (38).

### Testosterone Assay

Testosterone was measured by using ELISA kit purchased from Dia Metra, Giustozzi, Foligno (PG) Italy (LOT No: *DKO002*) as described earlier (31). The 25 μl of standard, control, or sample and 100 μl of diluted conjugate solution were added to each ELISA plate. The ELISA plate was then incubated at 37°C for 1 h with mild shaking. The wells were aspirated and washed several times with distilled water. After adding 100 μl of the tetramethyl benzidine (TMB) chromagen substrate to each well, ELISA plate was incubated at room temperature for 15 min in the dark. Finally, stop solution (100 μl) was added, and absorbance was recorded at 450 nm using a microplate reader. The standard curve ranged from 0.2 to 16 ng/ml (31).

#### Glucose Assay

Blood glucose was measured by the glucose oxidase method using a commercially available automated glucose analyzer (Span Diagnostics Ltd., Surat, Gujarat, India) with 10 μl of blood.

### Serum Triglyceride Assay and Adipose Tissue Triglyceride Content

Triglyceride in blood was measured using a commercially available colorimetric kit (GPO-Trinder) (Span Diagnostics Ltd., Surat, Gujarat, India), and TG in WAT was measured with minor modifications (41, 42). A 20% homogenate (w/v) of WAT was prepared in PBS. Then TG was extracted from the homogenate overnight in heptane:isopropanol (3:2) at 4°C. TG content was measured using a colorimetric kit (GPO-Trinder) from Span Diagnostics Ltd., Surat, Gujarat, India. Results are expressed as milligram TG per milligram protein.

### Glucose Uptake Assay: *In Vitro* Study

The media stored at −20°C was used for glucose uptake assay according to Roy and Krishna (38) and Banerjee et al. (16), who described earlier to determine the glucose by quantitative colorimetric method. The glucose concentration of the media was measured at the beginning as well as at the end of culture, and the difference between initial concentration of media and final concentration of media after the 24 h culture was taken as the amount of glucose uptake by the WAT or testis. Each group was run in triplicates. The intra-assay coefficient of variation (CV) was <7.5%.

### Statistical Analysis

Data were analyzed using one-way ANOVA followed by Bonferroni’s test using SPSS software 16 for Windows (SPSS Inc., Chicago, IL, USA). Correlation studies were performed to compare data from different groups. The differences were considered significant at the level of *p* < 0.05.

## Results

### Changes in Daily Food Intake

Table 2 shows the effect of *in vivo* administration of GnIH on food intake of adult male mice. Three different doses (20, 200 ng, and 2 μg/day) of GnIH administration caused a dose-dependent increase in food intake. A significant increase (*p* < 0.05) in food intake was noticed in mice treated with a high dose (2 μg/day) of GnIH for 8 days as compared with the control.

**Table 2:** **Effect of GnIH on average daily food intake in mice (*in vivo*)**.

Doses of GnIH	Daily food intake (g)			Average daily food intake (g)
	Day 1	Day 4	Day 8	
Control	18.4 ± 0.095	18.1 ± 0.170	17.8 ± 0.105	18.1 ± 0.173
20 ng/day	17.08 ± 0.04	16.2 ± 0.138	17.8 ± 0.084	17.05 ± 0.482
200 ng/day	18.01 ± 0.28	22.40 ± 0.44*	24.55 ± 0.183*	21.32 ± 1.69*
2 μg/day	22 ± 0.219*	24.55 ± 0.131*	25 ± 0.002*	23.85 ± 0.93*

*Values are mean ± SEM for five animals (daily food intake)*.

*Significantly different from controls (*p* < 0.05) by one-way analysis of variance (ANOVA) followed by Bonferroni’s test.

### Body Mass and Adipose Tissue Mass

The body mass showed significant (*p* < 0.05) increase by the treatment with moderate and high doses (200 ng and 2 μg/day) of GnIH as compared with the control. The adipose tissue mass also changed in response to the treatment with different doses (20, 200 ng, and 2 μg/day) of GnIH. The adipose tissue mass showed no significant change when treated with a low dose (20 ng/day) of GnIH, whereas it increased significantly (*p* < 0.05) in response to moderate and high doses of GnIH treatments as compared with the control (see Table 3).

**Table 3:** **Effect of GnIH on body mass, WAT mass, adipose tissue TG, testicular glucose, serum TG, and serum glucose mice (*in vivo*)**.

Doses of GnIH	Body mass (g)	White adipose tissue mass (g)	Tissue		Serum	
			Adipose tissue Triglyceride concentration (TG/mg protein)	Testicular glucose concentration (mg/mg testis wt)	Triglyceride level (mg/dl)	Glucose level (mg/dl)
Control	33.258 ± 0.23	2.121 ± 0.10	442.8 ± 5.34	4.4 ± 0.166	140.38 ± 1.58	43.86 ± 1.68
20 ng/day	33.022 ± 0.56	1.524 ± 0.04	179.8 ± 3.32*	3.8 ± 0.12	229.25 ± 2.01*	85.65 ± 1.49*
200 ng/day	34.236 ± 0.30	2.88 ± 0.08*	256.56 ± 10.86*	3.2 ± 0.176*	196.57 ± 1.43*	65.33 ± 1.85*
2 μg/day	38.89 ± 0.38*	3.22 ± 0.05*	602.65 ± 5.85*	2.5 ± 0.17*	93.36 ± 4.13*	31.65 ± 1.43*

*Values are mean ± SEM for five animals*.

*Significantly different from controls (*p* < 0.05) by one-way analysis of variance (ANOVA) followed by Bonferroni’s test.

### Circulating Triglyceride, Glucose, and Testosterone Levels

**Figure 1:**
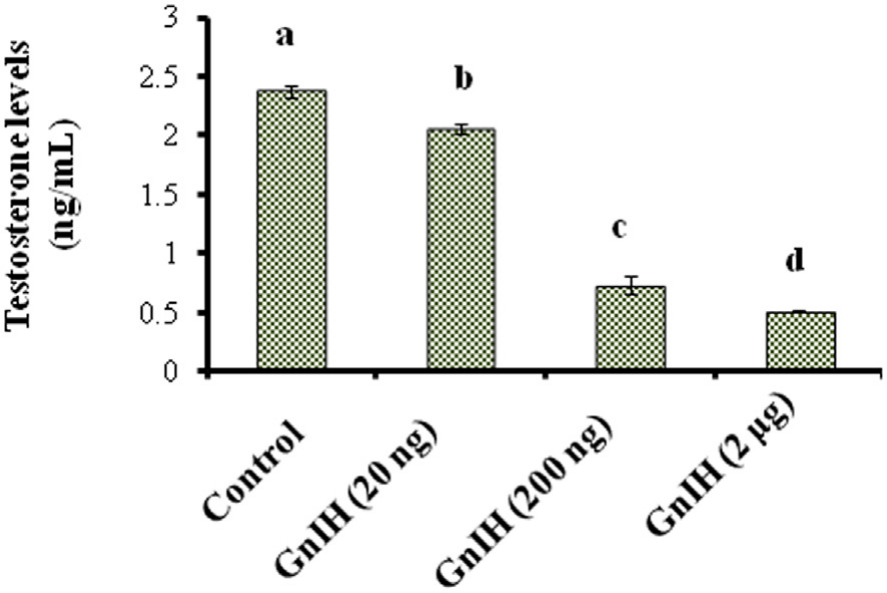
**The mice treated with different doses (20, 200 ng, and 2 μg/day) of GnIH on synthesis of testosterone in testis of mice**. GnIH showed significantly decreased (*p* < 0.05) synthesis of testosterone levels in dose-dependent manner as compare to control (a, *p* < 0.05).

The treatment with different doses (20, 200 ng, and 2 μg/day) of GnIH showed significant variation in circulating levels of TG s (*p* < 0.05) and glucose (*p* < 0.05). Both TG s and glucose levels were significantly (*p* < 0.05) increased in response to low and moderate doses (20 and 200 ng/day) of GnIH treatment as compared with the control. However, both TGs and glucose levels were significantly (*p* < 0.05) decreased with a high dose (2 μg/day) of GnIH in comparison to the control (Table 3). The treatment with different doses of GnIH showed a significant (*p* < 0.05) dose-dependent decline in circulating testosterone levels as compared with the control (Figure 1).

### Changes in the Level of Triglycerides and in the Expression of GLUT4 and GLUT8 and IR Proteins in the Adipose Tissue

The treatment with low and moderate doses (20 and 200 ng/day) of GnIH showed significant decrease (*p* < 0.05) in the TG level in the adipose tissue, whereas a high dose (2 μg/day) of GnIH treatment showed a significant increase (*p* < 0.05) in the TGs level in the adipose tissue as compared with the control (*p* < 0.05) (Table 3).

**Figure 2:**
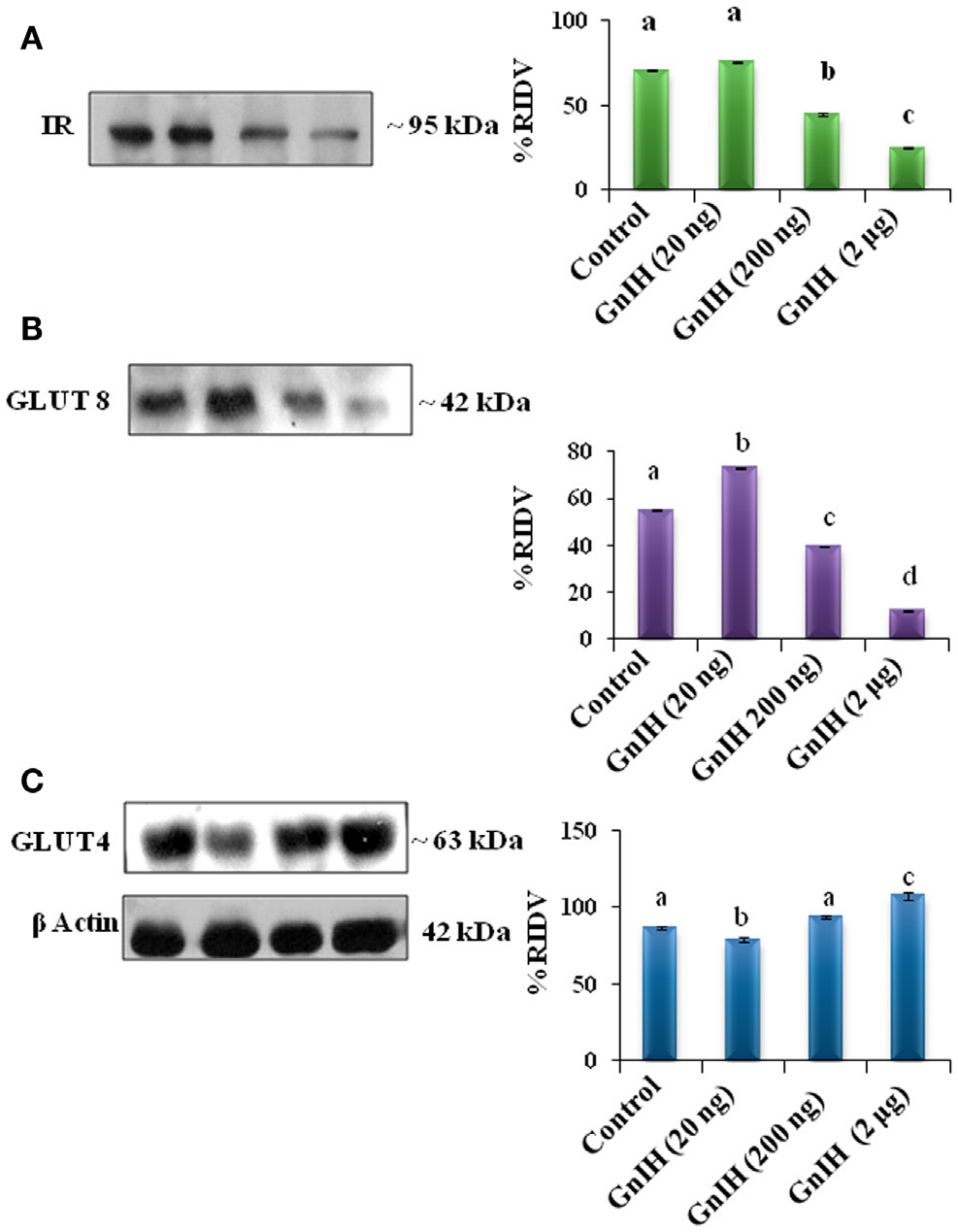
**The densitometric analysis of the Western blot showed the treatment of different doses (20, 200 ng, and 2 μg/day) of GnIH on expression of IR, GLUT8, and GLUT4 proteins in adipose tissue of mice**. **(A)** GnIH showed a dose-dependent significant (*p* < 0.05) decline in the expression of IR. Moderate and high doses of GnIH (200 ng and 2 μg/day) showed significant (b and c, *p* < 0.05) decrease in the expression of IR protein as compare with control (a, *p* < 0.05). **(B)** A low dose of GnIH (20 ng/day) showed significant (b, *p* < 0.05) increased in the expression of GLUT8 protein as compared to the control. However, moderate and high doses of GnIH (200 ng and 2 μg/day) showed significant (c and d, *p* < 0.05) decrease in the expression of GLUT8 protein as compare with control (a, *p* < 0.05). **(C)** A low dose (20 ng/day) of GnIH treatment showed significant (*p* < 0.05) decrease in the expression of GLUT4 whereas a high dose (2 μg/day) showed significant (*p* < 0.05) increase in the expression of GLUT4 as compared with the control in the adipose tissue. Values are mean ± SEM.

The treatment with low dose (20 ng/day) of GnIH showed significant (*p* < 0.05) increase in the expressions of IR and GLUT8 proteins in the adipose tissue as compared with the control. However, moderate and high doses (200 ng and 2 μg/day) of GnIH showed significant (*p* < 0.05) decreased expressions of IR and GLUT8 proteins as compared with the control (Figures 2A,B). A low dose (20 ng/day) of GnIH treatment showed significant (*p* < 0.05) decreased expression of GLUT4 whereas a high dose (2 μg/day) showed significant (*p* < 0.05) increased expression of GLUT4 as compared with the control in the adipose tissue (Figure 2C).

### Changes in the Level of Glucose and in the Expression of GLUT8 and IR Proteins in the Testis

The treatment with low and moderate doses (20 and 200 ng/day) of GnIH showed significant (*p* < 0.05) decrease in glucose concentrations in the testis as compared with the control. Whereas the treatment with a high dose (2 μg/day) caused a significant (*p* < 0.05) increase in glucose level in the testis as compared to treatment with the moderate dose (Table 3).

**Figure 3:**
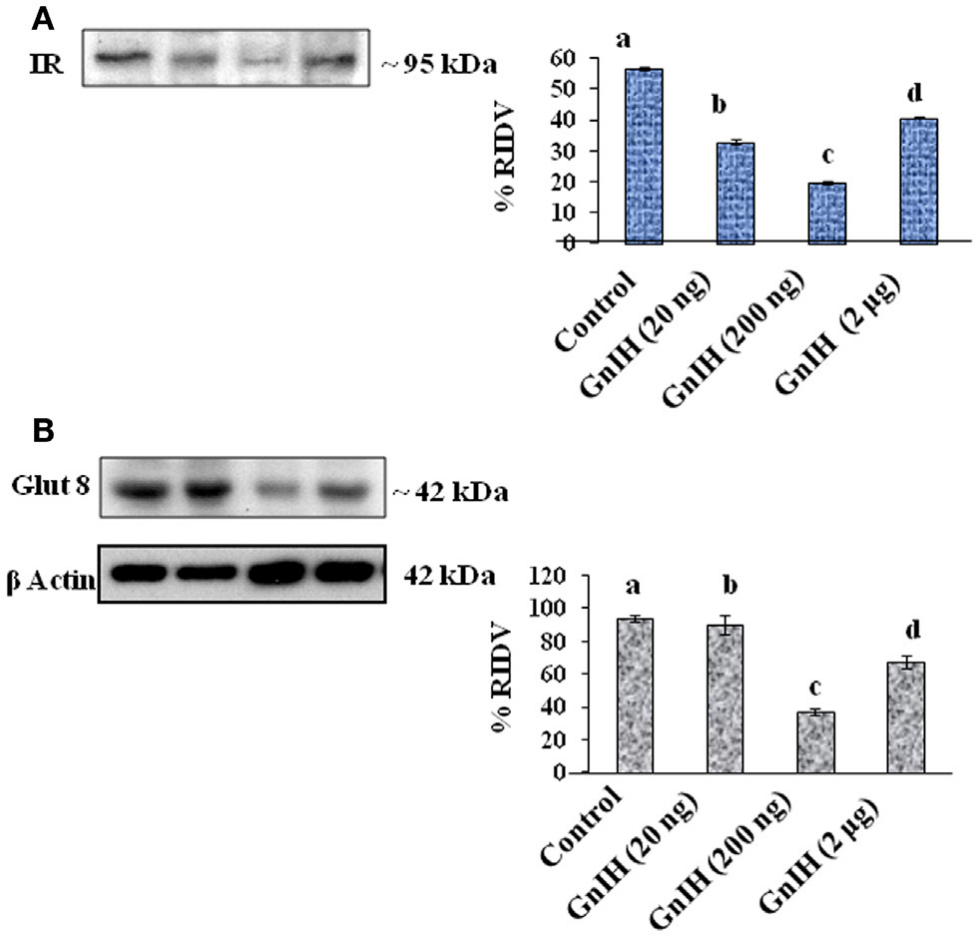
**The densitometric analysis of the Western blot showed the treatment of different doses (20, 200 ng, and 2 μg/day) of GnIH on expression of IR and GLUT8 proteins in testis of mice**. **(A)** The treatment with low and moderate doses (20 and 200 ng/day) of GnIH showed significant (b and c, *p* < 0.05) decrease in expression of IR protein as compared with the control, whereas high dose (2 μg/day) caused a significant (d, *p* < 0.05) increase in the expression of IR protein as compared with the control and moderate dose treatment. **(B)** The treatment with a low and moderate dose (20 and 200 ng/day) of GnIH showed a significant (b and c, *p* < 0.05) decrease in the expression of GLUT8 protein as compared with the control, whereas the treatment with a high dose (2 μg/day) caused a significant (d, *p* < 0.05) increase in the expression of GLUT8 protein as compared with the moderate dose treatment. Values are mean ± SEM.

The treatment with low and moderate doses of GnIH showed significant (*p* < 0.05) decrease in expression of IR protein in the testis as compared with the control, whereas the treatment with a high dose caused a significant (*p* < 0.05) increase in the expression of IR protein as compared with the moderate dose treatment (Figure 3A).

The treatment with a moderate dose (200 ng/day) of GnIH showed a significant (*p* < 0.05) decrease in the expression of GLUT8 protein in the testis as compared with the control, whereas the treatment with a high dose (2 μg/day) caused a significant (*p* < 0.05) increase in expression of GLUT8 protein in the testis as compared with the moderate dose treatment (Figure 3B).

### Effects of *In Vitro* Treatment of GnIH with or without LH in the Testis of Mice

#### Effect on Glucose Uptake

**Figure 4:**
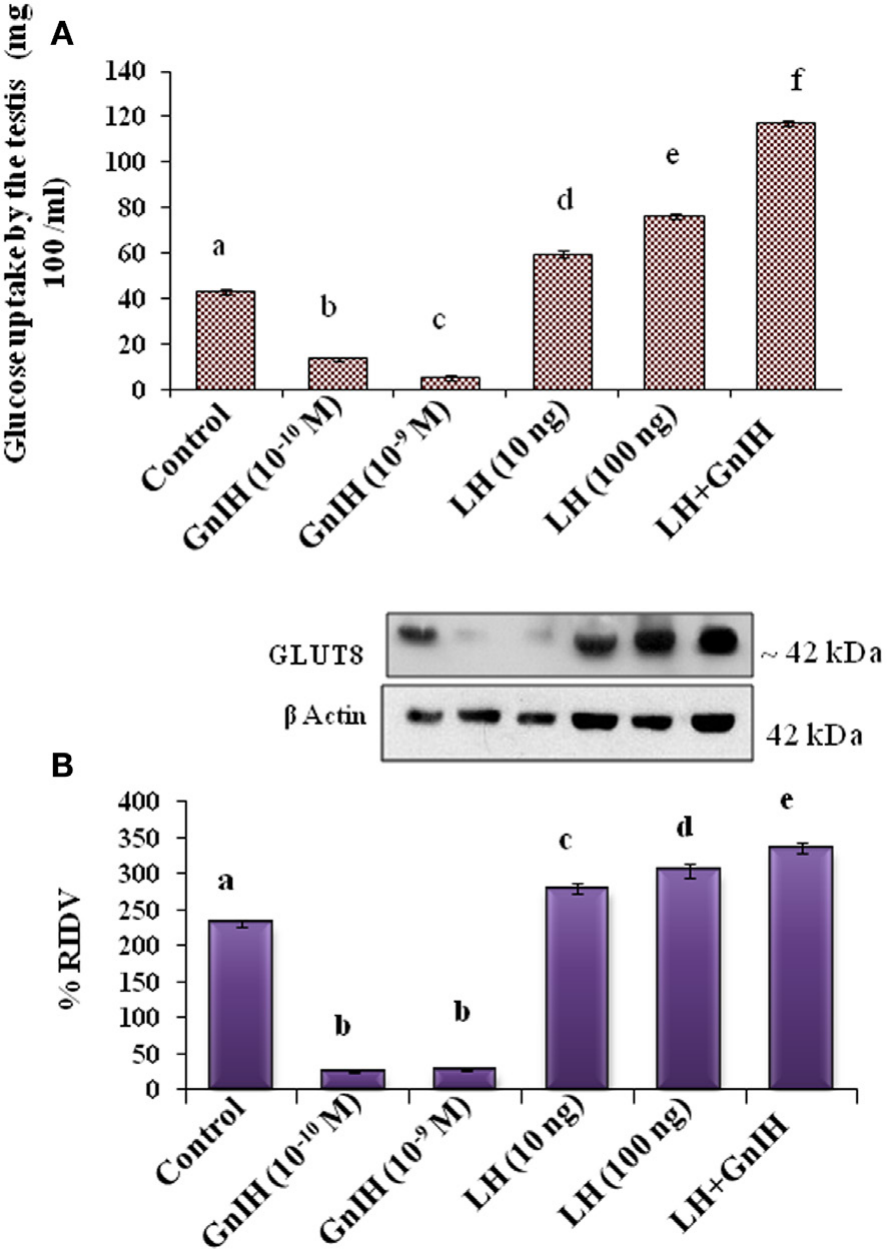
**The *in vitro* treatment of different doses (10^−10^ and 10^−9^M/ml) of GnIH with or without different doses (10 and 100 ng/ml) of LH showed effect on glucose uptake by the testis and expression of GLUT 8 protein**. **(A)** The treatment with low and high doses (10^−10^ and 10^−9^M/ml) of GnIH showed a dose-dependent suppression of glucose uptake in the testis. The treatment with low and high (10 and 100 ng/ml) doses of LH alone showed a dose-dependent significant (d and e, *p* < 0.05) increase in glucose uptake by the testis. A high dose of LH together with a high dose of GnIH showed a significant (f, *p* < 0.05) increase in uptake of glucose by the testis as compared with the control (a, *p* < 0.05). **(B)** The treatment with low and high doses (10^−10^ and 10^−9^M/ml) of GnIH showed a significant (b, *p* < 0.05) decrease in the expression of GLUT8 protein. The treatment with low and high doses (10 and 100 ng/ml) of LH showed a dose-dependent significant (c and d, *p* < 0.05) increase in the expression of GLUT8 protein. A high dose of LH with a high dose of GnIH showed a significant (f, *p* < 0.05) increase in the expression of GLUT8 protein in the testis as compared with the control (a, *p* < 0.05).

The *in vitro* treatment with low and high doses (10^−10^ and 10^−9^M/ml) of GnIH showed a dose-dependent suppression of glucose uptake in the testis. The treatment with low and high (10 and 100 ng/ml) doses of LH alone showed a dose-dependent significant (*p* < 0.05) increase in glucose uptake by the testis. The treatment with a high dose of LH together with a high dose of GnIH showed a significant (*p* < 0.05) increase in uptake of glucose by the testis as compared with the control (Figure 4A).

#### Effect on the Expression of GLUT8 Protein

The *in vitro* treatment with low and high doses (10^−10^ and 10^−9^M/ml) of GnIH showed a significant (*p* < 0.05) decrease in the expression of GLUT8 protein. However, the *in vitro* treatment with low and high doses (10 and 100 ng/ml) of LH showed a dose-dependent significant (*p* < 0.05) increase in the expression of GLUT8 protein. The *in vitro* treatment with a high dose of LH with a high dose of GnIH showed a significant increase in the expression of GLUT8 protein in the testis as compared with the control (Figure 4B).

### Effects of *In Vitro* Treatment of Insulin to the Adipose Tissue of Mice

#### Effects on the Expressions of IR, GLUT4, and AKT Proteins in the Adipose Tissue

**Figure 5:**
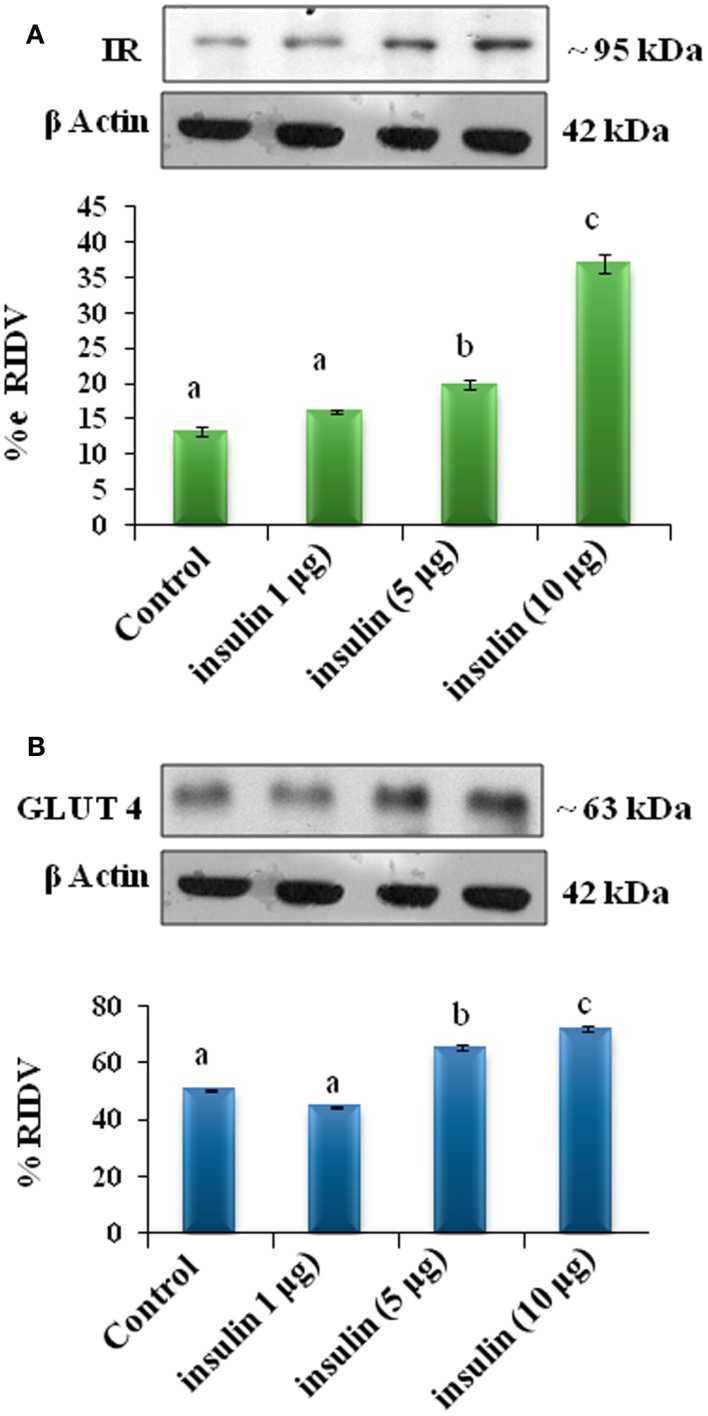
**The *in vitro* treatment with different doses of insulin (1, 5, and 10 μg/ml) on expression of insulin receptor and GLUT4 proteins in the adipose tissue**. The treatment of insulin showed dose-dependent significant (b, c and d, *p* < 0.05) increase in expression of IR **(A)** and (b and c, *p* < 0.05) GLUT4 **(B)** proteins in the adipose tissue as compared with the control (a, *p* < 0.05).

The adipose tissue treated *in vitro* with 1, 5, and 10 μg/ml doses of insulin showed dose-dependent significant (*p* < 0.05) increase in expression of IR, GLUT4 proteins in the adipose tissue as compared with the control (Figures 5A,B).

**Figure 6:**
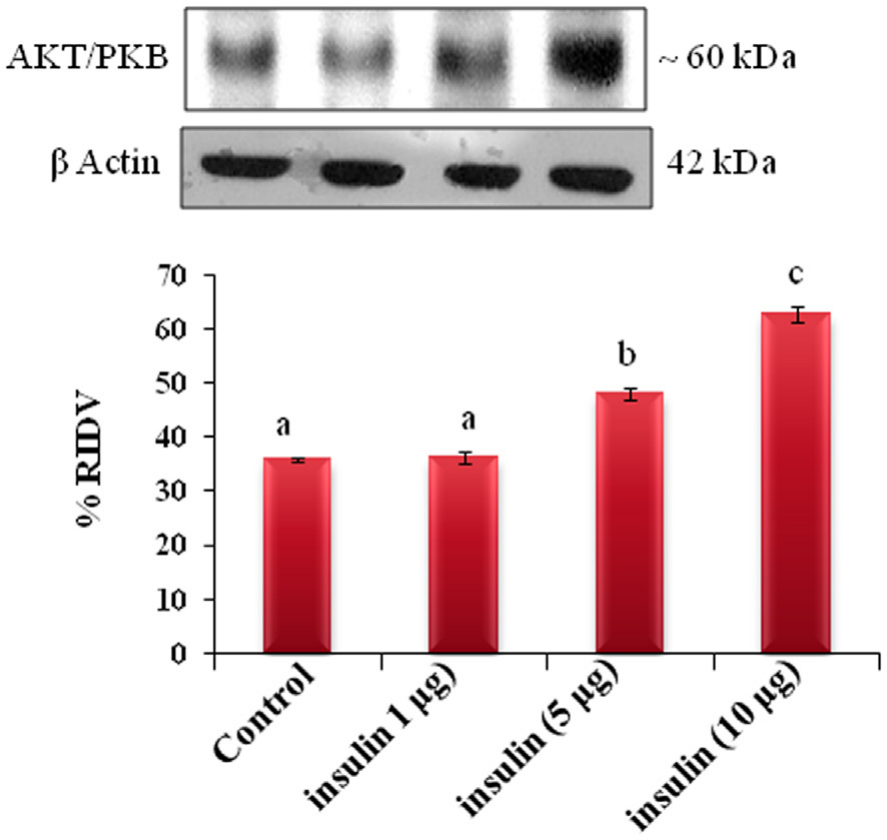
**The *in vitro* treatment of adipose tissue with different doses (1, 5, and 10 μg/ml) of insulin showed dose-dependent significant (b, c, and d, *p* < 0.05) increase in the expression of AKT/PKB protein in adipose tissue as compare to the control (a, *p* < 0.05)**.

The adipose tissue treated *in vitro* with different doses (1, 5, and 10 μg/ml) of insulin showed dose-dependent significant (*p* < 0.05) increase in the expression of AKT protein in adipose tissue as compare to the control (Figure 6).

### Effects of *In Vitro* Treatment of GnIH Either Alone Or Together with Insulin to the Adipose Tissue of Mice

#### The Expression of IR Protein

**Figure 7:**
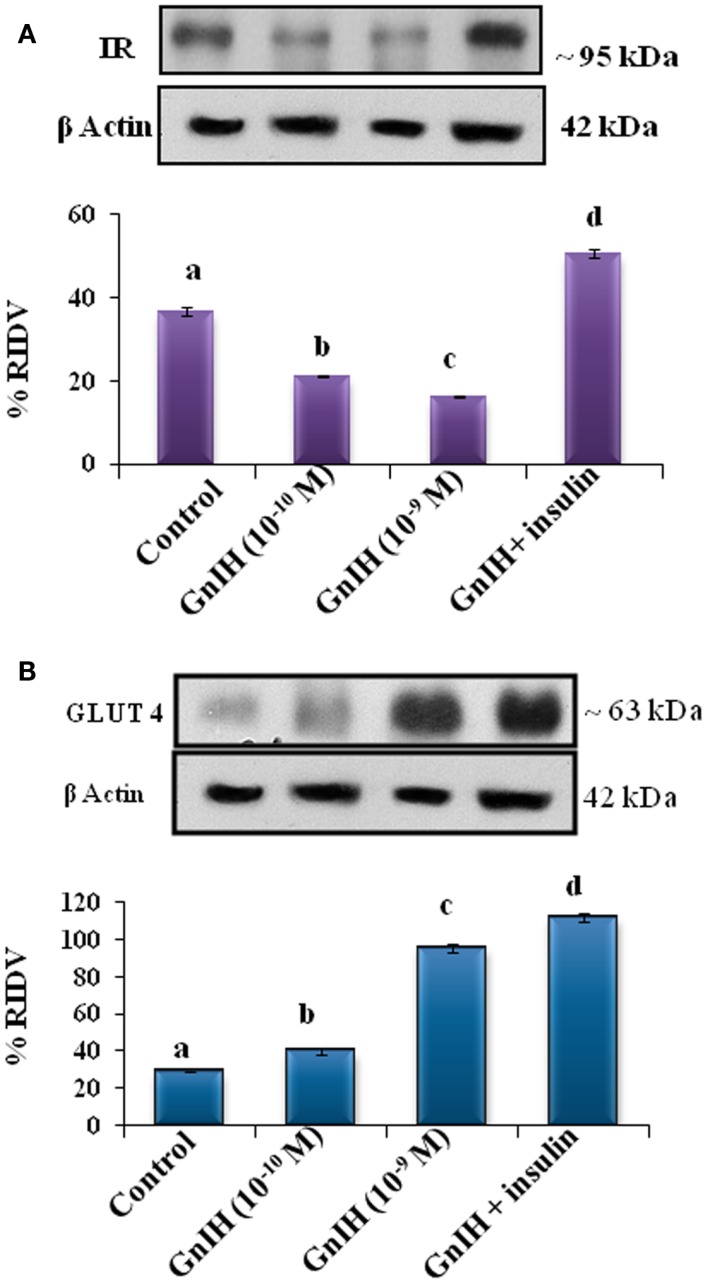
**The *in vitro* treatment of adipose tissue with different doses (10^−10^ M and 10^−9^ M/ml) of GnIH with or without insulin (10 μg/ml) showed (A) a dose-dependent (b and c, *p* < 0.05) significant decrease in expression of IR protein as compared with the control with (10^−10^ M and 10^−9^ M/ml) of GnIH**. The treatment with a high dose of GnIH together with insulin showed a significant (d, *p* < 0.05) increase in the expression of IR protein in as compared with the only GnIH-treated group. **(B)** The *in vitro* treatment of adipose tissue with different doses (10^−10^ and 10^−9^M/ml) of GnIH with or without insulin (10 μg/ml) showed a dose-dependent significant (*p* < 0.05) increase in the expression of GLUT 4 protein as compared with the control. The treatment with the high dose of GnIH together with insulin showed a significant (d, *p* < 0.05) increase in the expression of GLUT4 protein in the adipose tissue as compared with only GnIH-treated group.

The adipose tissue treated *in vitro* with different doses (10^−10^ and 10^−9^M/ml) of GnIH with or without insulin (10 μg/ml) showed a dose-dependent significant (*p* < 0.05) decrease in expression of IR protein as compared with the control. The treatment with a high dose of GnIH together with insulin showed a significant (*p* < 0.05) increase in the expression of IR protein in the adipose tissue as compared with the only GnIH-treated group (Figure 7A).

#### The Expression of GLUT4 Protein

The adipose tissue treated *in vitro* with different doses (10^−10^ and 10^−9^M/ml) of GnIH with or without insulin (10 μg/ml) showed a dose-dependent significant (*p* < 0.05) increase in the expression of GLUT 4 protein as compared with the control. The treatment with the high dose of GnIH together with insulin showed a significant (*p* < 0.05) increase in the expression of GLUT4 protein in the adipose tissue as compared with only GnIH-treated group (Figure 7B).

#### The Expression of AKT/PBK Protein

**Figure 8:**
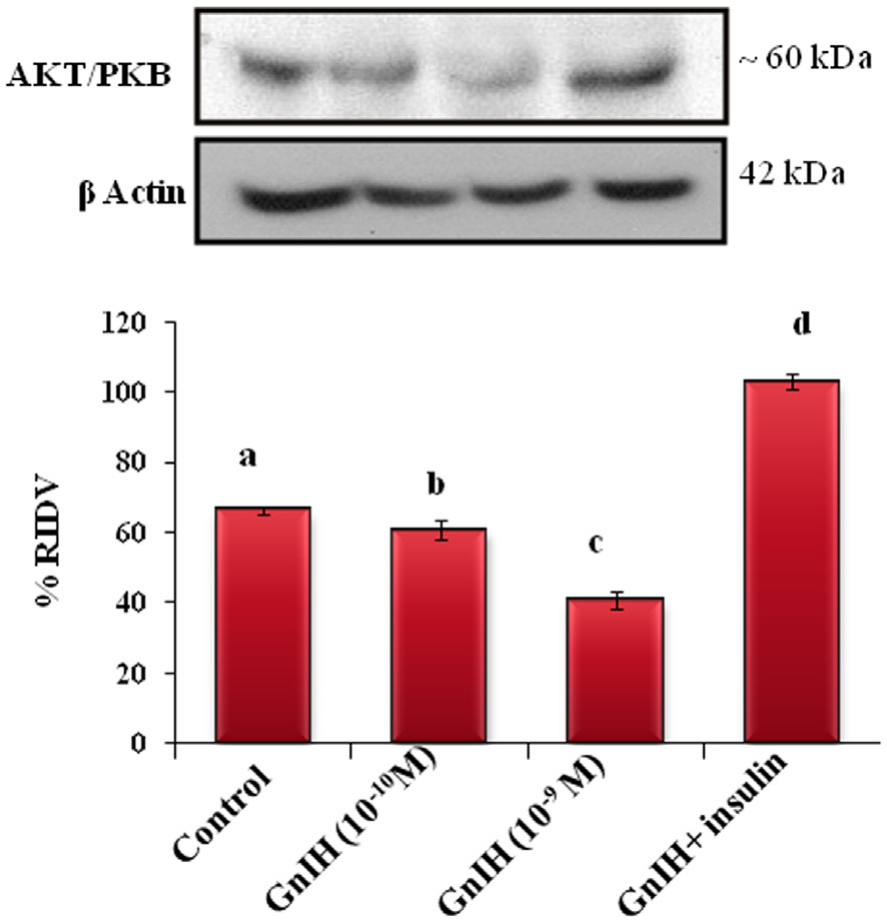
**The *in vitro* treatment of adipose tissue with different doses (10^−10^ and 10^−9^M/ml) of GnIH alone showed a dose-dependent significant (b and c, *p* < 0.05) decrease in the expression of AKT/PKB protein as compared with the control**. The treatment with a high dose of GnIH together with insulin showed a significant (d, *p* < 0.05) increase in the expression of AKT/PKB protein in the adipose tissue as compared with the only GnIH-treated group. Values are mean ± SEM.

The adipose tissue treated *in vitro* with different doses (10^−10^ and 10^−9^M/ml) of GnIH alone showed a dose-dependent significant (*p* < 0.05) decrease in the expression of AKT protein as compared with the control. The treatment with a high dose of GnIH together with insulin showed a significant (*p* < 0.05) increase in the expression of AKT protein in the adipose tissue as compared with the only GnIH-treated group (Figure 8).

### Correlation Study

The results of correlation study studies are summarized in Table 4. A significant (*p* < 0.05) correlation was found between changes in circulating testosterone levels, glucose levels, and TG levels with changes in the rate of expression of GLUT4, GLUT8, and IR in adipose tissue and testis of mice treated with GnIH.

**Table 4:** **Correlation studies of GnIH treatment between the different parameters in adipose tissue and testis (*in vivo*)**.

	Adipose tissue TG	Serum TG	Serum glucose	Testicular glucose level	Testosterone level	Adipose tissue			Testis	
						GLUT4	GLUT8	IR	GLUT8	IR
Adipose tissue TG		*r* = −0.99, *p* < 0.05	*r* = −0.97, *p* < 0.05	NS	NS	*r* = 0.89, *p* < 0.05	*r* = −0.67, *p* < 0.05	*r* = −0.57, *p* < 0.05	NS	*r* = 0.57, *p* < 0.05
Serum TG			*r* = 0.98, *p* < 0.05	NS	NS	*r* = −0.91, *p* < 0.05	*r* = 0.67, *p* < 0.05	*r* = 0.57, *p* < 0.05	NS	*r* = 0.56, *p* < 0.05
Serum glucose				NS	NS	*r* = −0.93, *p* < 0.05	*r* = 0.63, *p* < 0.05	NS	NS	*r* = −0.56, *p* < 0.05
Testicular glucose level					*r* = 0.94, *p* < 0.05				*r* = 0.63, *p* < 0.05	NS
Testosterone level									*r* = 0.82, *p* < 0.05	*r* = 0.56, *p* < 0.05
GLUT4							*r* = −0.97, *p* < 0.05	*r* = −0.94, *p* < 0.05	NS	NS
GLUT8								*r* = 0.99, *p* < 0.05	NS	*r* = 0.78, *p* < 0.05

*NS, not significant*.

*Values are significantly different at *p* < 0.05*.

## Discussion

For the first time, the results of present study provide the experimental proof suggesting an active participation of GnIH in increased food intake and glucose and TGs uptake in adipose tissue results in fat accumulation and increased body mass whereas in testis, GnIH suppressed glucose uptake resulting in decreased testosterone synthesis. In the present study, three different doses of GnIH were used, and the results showed dose-dependent changes in majority of the studies. But treatment with the high dose of GnIH (2 μg/day) showed a significant variation as compared with the control, thus the results of high dose of GnIH treatment are generally considered for discussion.

The mice treated *in vivo* with GnIH showed both dose- and duration-dependent increase in food intake. High dose of RFRP-3 (2 μg/day) showed increased food intake on day 1 itself as compared with the control, whereas moderate dose of RFRP-3 (200 ng/day) showed significant increase in food intake on day 4. The lower dose of RFRP-3 did not show any significant increases in food intake until day 8 (Table 2). Thus moderate and high doses of GnIH induced a significant increase in food intake as compared to the control, and it is therefore considered that GnIH increases food intake in mice. In addition, the mice treated *in vivo* with GnIH for 8 days showed increased accumulation of adipose tissue and body mass as compared to the control. These findings are in agreement with earlier observations in chicks and rat (32, 43) and suggest that GnIH is one of the orexigenic peptide in mammals. An earlier study has demonstrated the presence of RFRP-3 receptor (GPR147) in the adipose tissue (44). The GnIH containing neurons was shown in contact with the neurons containing neuropeptides, such as neuropeptideY, pro-opio melanocortin, orexin, and melanopeptide, which are known modulators of nutritional changes (28, 45, 46). In an earlier study, where rat was treated with GnRH agonist showed a dose-dependent gain in the body mass (47). It was suggested that GnRH agonist-induced increase in the body mass might be due to neuropeptideY- and/or opioidpeptide-mediated increase in feeding activity in the rats (48). Accordingly, in the present study, GnIH may act as a modulator of energy homeostasis association with other neuropeptides in the mice.

Interestingly, in the present study, *in vivo* treatment of GnIH showed dose-dependent increase in the expression of GLUT4 protein, but significant decline in the expression of GLUT8 and IR proteins in the adipose tissue. GLUT4, a major isoform of GLUT, is normally found in the insulin-responsive tissue, such as the striated muscle and adipose tissue, and plays a crucial role in whole body glucose homeostasis. It is well known that insulin upregulates the expression of GLUT4 protein (49, 50). Besides GLUT4, GLUT8, and GLUT12 are other GLUTs regulated by insulin (51). GLUT8 is mainly expressed in the striated muscle, adipose tissue, testis, blastocyst, brain, liver, and kidney (52, 53). The upregulation of GLUT4 by GnIH in the adipose tissue is unique findings of this study. Despite increased expression of GLUT4, GnIH treatment caused dose-dependent downregulation of GLUT8 and IR proteins in the adipose tissue. In adipose tissue, expression of GLUT4 is under regulation of insulin, but regulation of GLUT8 is not known. This study also showed a significant positive correlation between changes in expression of IR with GLUT8 protein but showed a negative correlation between the changes in expression of IR with GLUT4 protein in the adipose tissue treated *in vivo* with GnIH. This suggests that in adipose tissue, expression of GLUT8 protein changes with IR level. Furthermore, the *in vitro* study confirmed the *in vivo* observations of GnIH-induced upregulation of GLUT4 together with the downregulation of IR proteins in the adipose tissue. These observations thus suggest that GnIH increases glucose uptake in the adipose tissue stimulating expression of GLUT4.

The *in vivo* treatment with high dose of GnIH showed significantly increased synthesis of TGs in adipose tissue. GnIH-induced increased expression of GLUT4 suggests increased uptake of glucose into adipose tissue, which subsequently may get converted into TG and free fatty acid, the storage form of nutrients in the adipocytes. The mice treated with GnIH *in vivo* showed dose-dependent significant decline in the circulating TG and glucose levels. Simultaneously with decline in circulating TG levels, this study showed significant increase in the TG level in the adipose tissue as compared with the control. The significant inverse correlation between changes in TG level in the serum verses adipose tissue suggest that GnIH may be responsible for increased transport of TGs from serum to adipose tissue. This consequently may be responsible for increased accumulation of fat in the adipocytes and consequently increase in body mass.

The mice treated *in vivo* with GnIH showed a dose-dependent significant (*p* < 0.05) decline in the level of testosterone as compared with the control. The earlier study on hamster testes revealed the expression of RFRP-receptor (GPR147). Thus, suggesting autocrine or paracrine role of RFRP in testis (54). The GnIH treatment *in vivo* also showed a significant decline in the expression of GLUT8 and IR proteins together with decreased concentration of glucose in the testis. This finding thus suggests that the mice treated with GnIH caused reduced availability of glucose to the testis; this consequently may be responsible for the decreased testosterone synthesis. This finding further confirms our recent study that the decreased availability of glucose to the testis resulted in the decreased steroidogenesis (16). It has earlier been demonstrated that glucose availability also modulates the level of the steroidogenic enzyme nicotinamide adenine dinucleotide phosphate (NADPH) in the testis (55). The *in vivo* treatment of GnIH also caused suppressed expression of IR protein in the testis. Since insulin directly affects testicular steroidogenesis *via* the induction of dosage-sensitive sex reversal, adrenal hypoplasia critical region, on chromosome X, gene 1 (DAX-1) in Leydig cells (56), it possible that GnIH may inhibit steroidogenesis by downregulating IR in the testis.

Similar to *in vivo* study, the testes treated *in vitro* with GnIH also showed significant decreased expression of GLUT8 protein, decreased concentration of glucose, and significant decline in the testosterone synthesis. These findings thus suggest that the GnIH-induced decreased uptake of glucose may be directly responsible for the inhibition of testicular steroidogenesis. This study confirmed the earlier finding that Leydig cells culture in the absence of glucose can synthesis testosterone at very low rate (16, 57). The testis treated *in vitro* with LH either alone or together with GnIH showed a stimulatory effect on the glucose uptake together with the increased expression of GLUT8 protein and increased synthesis of testosterone. This study suggests that in presence of physiological concentration of LH, inhibitory effect of GnIH is suppressed. Our earlier studies have shown that LH or hCG increases glucose uptake by increasing GLUT8 expression in the testis (16). Thus in the physiological condition, when LH in sufficient concentration exist in the plasma, GnIH may facilitate glucose uptake by promoting GLUT8 in testis.

To investigate the signaling pathway of GnIH and insulin-mediated glucose uptake, AKT/PKB levels were evaluated in the adipose tissue. The adipose tissue from mice treated with different doses of GnIH with or without insulin *in vitro* showed a significant variation in the expression of AKT/PKB protein together with the expression of GLUT4 and IR proteins. The GnIH-induced changes in GLUT4 levels showed no significant correlation with the changes in AKT/PKB levels, but correlated significantly with the changes in IR protein. Furthermore, the insulin-induced changes in GLUT4 and IR levels correlated significantly with the changes in AKT/PKB levels in the adipose tissue. Although AKT/PKB is involved in many signaling pathways including glucose trafficking (58), this finding suggests that AKT/PKB may function as a signaling molecule in the insulin-induced GLUT4 (59) and IR expression as well as in the GnIH-induced IR expression in the adipose tissue. But AKT/PKB may not function as a signaling molecule in the GnIH-induced changes in the expression of GLUT4 protein in the adipose tissue.

In brief, the results of this study showed differential effect of GnIH-induced changes in nutrient levels of the adipose tissue and testis of mice. The *in vivo* treatment with GnIH showed the increased expression of GLUT4 together with increased uptake of TGs in the adipose tissue, which in turn resulted in the increased accumulation of fat into WAT and increase in the body mass. On the contrary in the testis, GnIH treatment *in vivo* caused downregulation of GLUT8 expression resulting in the decreased uptake of glucose, which in turn resulted in the decreased synthesis of testosterone. The mice treated with GnIH also showed the decreased expression of IR protein in the testis. The decreased level of IR may be responsible for the decreased testicular activity in the mice. The *in vitro* treatment of GnIH to the adipose tissue showed the increased expression of GLUT4 suggesting the increased uptake of glucose, despite decrease in IR. The *in vitro* treatment of GnIH to the testis showed decreased expression of GLUT8 protein resulted in decreased uptake of glucose and testosterone synthesis. These findings thus suggest that GnIH increases nutrients (glucose and TGs) uptake in the adipose tissue resulted in accumulation of fat, whereas in the testis GnIH suppresses the glucose uptake resulted in the decreased testosterone synthesis in mice. This study further showed that the insulin-induced upregulation of GLUT4 expression may be PKB/AKT mediated, whereas GnIH-induced upregulation of GLUT4 is not mediated through PKB/AKT mechanisms. On the basis of present study, GnIH appears to serve an important role in determining the level of fat accumulation in the adipose tissue and accordingly modulating the reproductive strategies to be adopted by the animal. Further studies are required to find out whether the GnIH is associated with decline in testosterone as observed during diabetes mellitus type II, aging, or subfertility in men.

## Author Contributions

SA executed experimental design and analyze the result and manuscript preparation, AK analyze the result and edited the manuscript, and KT edited the whole manuscript with important suggestions.

## Conflict of Interest Statement

The authors declare that the research was conducted in the absence of any commercial or financial relationships that could be construed as a potential conflict of interest.
